# Individual differences in working memory capacity moderate effects of post-learning activity on memory consolidation over the long term

**DOI:** 10.1038/s41598-020-74760-z

**Published:** 2020-10-21

**Authors:** Markus Martini, Robert Marhenke, Caroline Martini, Sonja Rossi, Pierre Sachse

**Affiliations:** 1 University of Innsbruck, 6020 Innsbruck, Austria; 2 Medical University of Innsbruck, 6020 Innsbruck, Austria

**Keywords:** Working memory, Human behaviour

## Abstract

Similar to sleeping after learning, a brief period of wakeful resting after encoding new information supports memory retention in contrast to task-related cognition. Recent evidence suggests that working memory capacity (WMC) is related to sleep-dependent declarative memory consolidation. We tested whether WMC moderates the effect of a brief period of wakeful resting compared to performing a distractor task subsequent to encoding a word list. Participants encoded and immediately recalled a word list followed by either an 8 min wakeful resting period (eyes closed, relaxed) or by performing an adapted version of the d2 test of attention for 8 min. At the end of the experimental session (after 12–24 min) and again, after 7 days, participants were required to complete a surprise free recall test of both word lists. Our results show that interindividual differences in WMC are a central moderating factor for the effect of post-learning activity on memory retention. The difference in word retention between a brief period of wakeful resting versus performing a selective attention task subsequent to encoding increased in higher WMC individuals over a retention interval of 12–24 min, as well as over 7 days. This effect was reversed in lower WMC individuals. Our results extend findings showing that WMC seems not only to moderate sleep-related but also wakeful resting-related memory consolidation.

## Introduction

### Wakeful resting and memory consolidation

The period following encoding of new information plays an essential role in memory retention^1–4^. It is assumed that any cognitive task performed following learning of new information affects retention of the previously-learned information^5^. For instance, Dewar, Cowan, and Della Sala^3^ found that participants recalled fewer words from a word list when they were required to search for errors in pictures, detect tones, or watch movies following encoding of a word list compared to a condition in which they were required to rest for several minutes with their eyes closed. During states in which attentional demands of new information are low, like wakeful resting, memory retention is supported, while high attentional engagement following learning, like performing a cognitive task, has detrimental effects on memory retention. This so-called resting effect was found in different populations (children, younger and older adults^6–9^), with different encoding material (verbal, visuospatial^3,10, 11^) and distractor tasks (spot-the-difference task^7^, vocabulary learning^12^) as well as retention intervals (minute^3^; days^7^; for conflicting results, see^13,14^).

One assumption why wakeful resting supports memory retention refers to the neuroscientific concept of memory consolidation. Memory consolidation describes a family of neural processes that stabilise and redistribute information in our brain^15,16^. States of reduced interference, in which no new memories are encoded following learning, have been found to facilitate memory consolidation. These states include slow-wave sleep, inhibited long-term potentiation using NMDA antagonists or other amnesia-inducing drugs, or short periods of wakeful resting^17^. These studies indicate that the process of memory consolidation takes time. This view is based on findings that newly-learned information continues to be processed ‘off-line’ following learning^15^. For instance, Tambini et al.^18^ found enhanced functional connectivity between hippocampus and neocortical regions during a resting period immediately following an associative memory task, compared to pre-task baseline resting connectivity. The finding that memories are particularly vulnerable to disruption in the first few minutes following learning suggests that this period of time is particularly important for memory consolidation^19,20^. Neuroscientific studies suggest that neuronal activity during states of sleep and wakeful resting share similarities^21^. For instance, Brokaw et al.^22^ found a positive correlation between retained memories of short stories and increased slow oscillatory activity (< 1 Hz), in concert with reduced alpha activity (8–12 Hz) during a wakeful resting state—cortical activity that is also found during sleep.

### Working memory and its interaction with long-term memory

Working memory (WM) represents a central construct in the cognitive architecture of the mind. It describes a cognitive system consisting of several processes relevant for higher-order, goal-related cognition. The capacity of WM is limited. WM capacity (WMC) refers to a person´s individual capacity for WM as the maximum amount of information that can be temporarily held in mind. WMC is positively correlated with encoding of new information^23^, vocabulary learning^24^, proactive and retroactive interference^25^, language comprehension^26^, and fluid intelligence^27^. Interindividual differences in WMC can arise through, among others (i) differences in the amount of information that can be maintained in a temporarily accessible state under conditions of interference (from external, e.g. a second task, or internal resources, e.g. thoughts), and (ii) building, maintaining and rapidly updating information bindings^28,29^ (e.g. list items to list position). Long-term memory plays an essential role in most WM theories^30–33^. For instance, Cowan^31,34^ proposed a model of WM consisting of long-term memory, a subset of memory elements which are in an activated state, and within that, a smaller subset of memory elements which are maintained in the focus of attention. How WM and long-term memory interact is a matter of debate. Studies showed that long-term memory encoding is facilitated by maintaining the to-be remembered information in WM^35–37^ and when information is more deeply processed^38–40^. Long-term memory encoding is impaired when especially early stages of WM maintenance are disturbed^41^ or WM load during encoding is high^42^. Additionally, Unsworth and Engle^33^ proposed that interindividual differences in WMC reflect not only differences in the ability to hold information in an active state but also differences in the ability to retrieve information from long-term memory^33,43,44^. In line with this view, Unsworth et al.^44^ found that individuals with high WMC retrieved more animal names in a category fluency task than those with low WMC.

### The present study

Fenn and Hambrick^45^ found that WMC is related to sleep-dependent declarative memory consolidation. In their study, participants in a waking condition and sleeping condition learned word pairs and recalled them at the end of a training phase and again 12 h later. Their central finding was that memories for word pairs improved after a period of sleep and that this increase in memory performance after sleep was positively related to participants’ WMC. Fenn and Hambrick speculated that higher WMC individuals created stronger associations between words at encoding and consequently showed greater reactivation during sleep resulting in an increased delayed memory performance (see also^46,47^). Together with the findings reported earlier, this indicates that higher and lower WMC individuals process information subsequent to learning more efficiently^48^. Based on findings that (i) wakeful resting supports the retention of new memories^6,7, 10–12,22,49,50^, (ii) WMC is associated with sleep-related declarative memory consolidation^45–47^ and that (iii) neural processes found during closed eye, wakeful resting resemble those found in sleep^7,18,22,51,52^, we investigated whether interindividual differences in WMC moderate the beneficial effect of a brief period of wakeful resting after learning compared to distraction on memory retention.

We used a study design applied in a number of previous wakeful resting studies^7,8,13,50^. Each participant learned two word lists. After encoding and immediate recall of the first word list, participants rested for 8 min and, after encoding and immediate recall of the second word list, they performed an attention (d2) task for 8 min. Word lists and delay conditions were counterbalanced. Participants recalled both word lists at the end of the experimental session and again after 7 days. Participants’ WMC was measured with a frequently applied WM task, the operation span (OSPAN^44,45^; for details, see Method section). Based on the outlines above, we hypothesised that (i) more words will be retained in the wakeful resting condition compared to the d2 condition and (ii) WMC will positively interact with the resting effect.

## Materials and methods

### Participants

Ninety-eight university students (65 female, 33 male; mean age = 21.96 years, age range = 18–42 years) participated in this experiment in exchange for course credit. This research was approved by the Board for Ethical Questions in Science of the University of Innsbruck and all procedures adhered to the appropriate ethical principles for research with humans. All participants were briefed and provided their informed consent in writing prior to their participation.

### Materials and procedure

The experiment included two testing sessions, Session 1 and Session 2, which were separated by 7 days^7,10,50^.

#### Session 1

Session 1 included two successive encoding conditions. All instructions were collectively given before the respective word-learning phase. Each encoding condition consisted of a 2-min buffer filled with ambient music^53^ followed by the visual presentation of one of two word lists taken from parallel test forms (A and C) of the verbal learning and memory test^54^. Each word list consisted of 15 mono- and bi-syllabic, highly familiar German nouns (e.g. ‘Trommel’ [drum], ‘Vorhang’ [curtain], ‘Glocke’ [bell]). Words were semantically unrelated within as well as between the word lists, did not rhyme and were presented sequentially in the middle of the computer screen for 500 ms with an inter-stimulus-interval of 1500 ms. Participants were instructed to remember as many words as possible for a subsequent recall test. After the word presentation, an immediate free recall test took place. Participants were instructed to write down as many words as possible, irrespective of the presentation order. Recall time was limited to 1 min.

Immediate recall was followed by an 8-min delay condition, which participants spent either wakefully resting or performing a distractor task. During the wakeful resting condition, participants were asked to wakefully rest with their eyes closed in the darkened room for 8 min. The experimenter rested with the participants. During the distractor phase, participants performed an adapted version of the d2 Test of Attention^55^. The d2 represents an attention and concentration performance test, in which participants see several rows of the letters ‘d’ and ‘p’ with a varying number of marks (between one and four) above and/or below the letters. Participants were required to cross out as many d’s with two marks as possible in 20 s per row. We employed this task, as it requires sustained focused attention and has few overlapping elements with the word lists, minimising the effects of retrieval competition in the subsequent recall test. The task was adapted from the original test by adding a second page with another 10 of the constantly repeating three rows of letter-mark combinations, so the duration of the task would exactly match the 8-min wakeful resting delay.

Following the example of Brokaw et al.^22^ we assessed participants’ mental activity during the wakeful resting condition and d2 condition. Participants were questioned (in written form) how often during the preceding 8-min interval they were engaged in six predefined categories of mental activity, including 'thinking about the past' (four items: I've been thinking about something that's in the past/that happened earlier today/in the past seven days/in the last weeks or years), 'thinking about the future' (four items: I've been thinking about something that's in the future/that might happen later today/in the next seven days/in the next weeks or years), 'I let my mind wander’, 'my mind was blank', 'I've been thinking about what I'm doing right now', and one item assessing intentional rehearsal of the word list ('I tried to rehearse the words I had learned before'). Answers were given on a Likert scale ranging from 1 (not at all) to 6 (very often).

**Figure 1:**
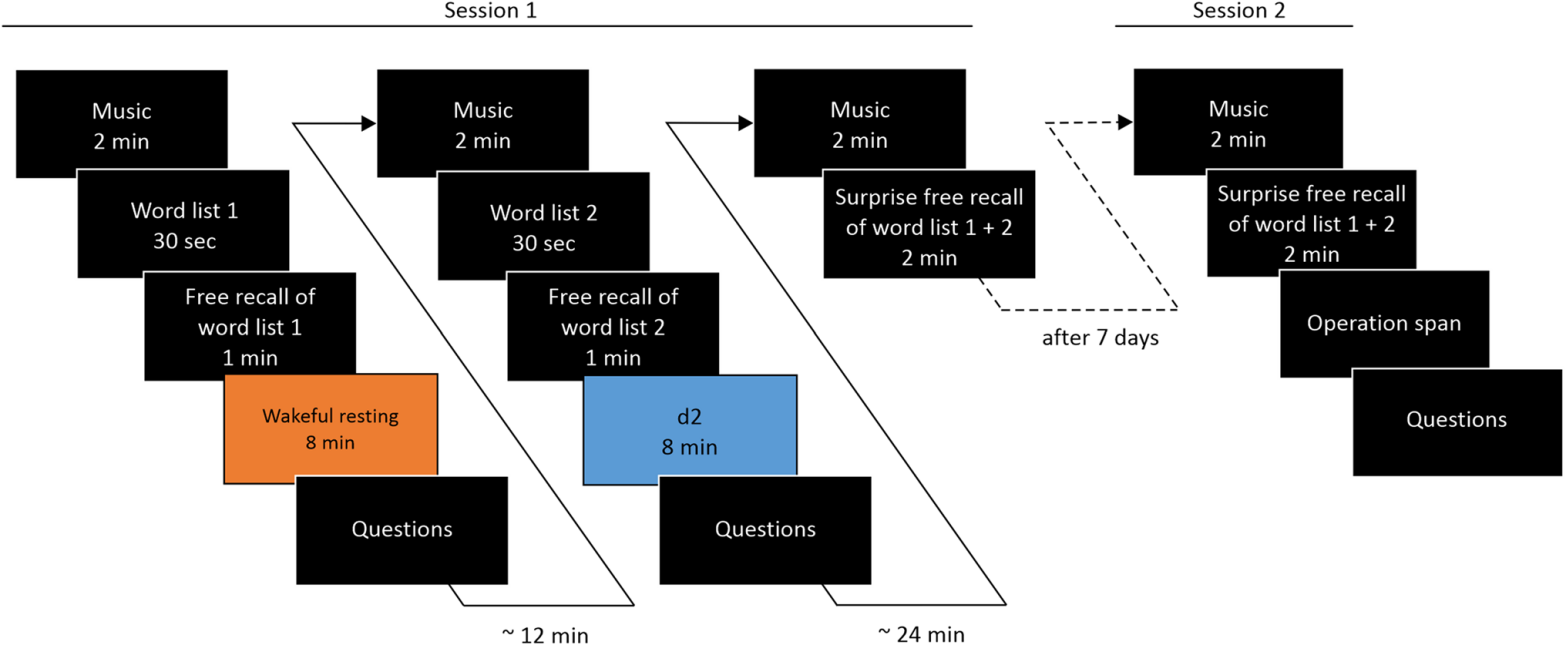
Experimental procedure. Participants encoded two word lists (2 × 15 words). The critical manipulation occurred after the immediate free recall test of the respective word list, where participants either wakefully rested or performed the d2 task. The order of the delay conditions (wakeful resting, d2) and word lists (lists 1 and 2) were counterbalanced across participants. A surprise free-recall test was conducted at the end of Session 1 and after 7 days in Session 2.

After another 2-min buffer filled with ambient music, a not-instructed surprise free recall test for both word lists occurred (Fig. 1). Participants were instructed to recall as many words as possible from both word lists, in any order they wanted. Participants noted the words on a blank sheet of paper. The order of the two delay conditions (rest, d2) and the two word lists were counterbalanced across participants.

#### Session 2

In Session 2, participants were again asked to recall both word lists. Before recall, a 2-min buffer filled with ambient music was included (Fig. 1). After recall, participants’ WMC was assessed with the automated OSPAN task^56,57^ (see also^44,45^). In this task, participants were asked to maintain or recover mental access to randomly selected capital letters in the right order while intermittently verifying solutions to simple math problems (by pressing the right or left arrow keys for either 'yes' or 'no' on a keyboard). Participants first solved a math problem, saw a letter, solved another math problem and saw another letter etc. Letters appeared 200 ms after each processing item and stayed on the screen for 250 ms. This math-letter sequence was repeated three to seven times for each trial, without any hint to the length of the trial. Each sequence length was shown three times in random order. Between trials, participants were shown accuracy feedback of the processing task. They were warned during the task instructions, that their data only could be used in the study if they achieved an accuracy rating of at least 85%. Following the processing-letter pairs, twelve letters appeared in a grid formation on screen next to a checkbox. Participants were asked to select, via mouse click, the presented letters in the order displayed earlier; the letters were shown in the checkbox. Participants were instructed to select a 'blank' button on the selection grid for forgotten letters, to preserve item order. Before beginning the actual OSPAN task, participants completed four practice trials of the memory-only, then 15 processing-only, and three combined practice trials. To prevent trading off between the processing and memorial part of the task, an individualised response deadline for the processing part (*M* + 2.5 *SD*s) was calculated during a processing-only practice trial. At the end of the session, participants were asked whether they had expected the surprise recall test.

Scoring of word lists. Participants’ performance for each word list recall was measured by the number of words correctly recalled (maximum of 15 words per word list).

WMC scoring. The performance measure for the OSPAN task was the partial storage score, which is the sum of items recalled in the correct serial position, regardless of whether the entire trial was recalled correctly^48^.

## Results

The alpha level was set at < 0.05. Analyses are based on the correctly recalled words per recall time (immediate, after 12–24 min, after 7 days) and delay condition (wakeful resting, d2). Five participants were excluded from all analyses since they did not appear to Session 2 after 7 days (*n* = 3) or had corrupted WMC data (*n* = 2).

### Memory performance without WMC as covariate

Immediate recall performance did not differ between the wakeful resting (*M* = 10.48, *SD* = 2.69) and d2 (*M* = 10.38, *SD* = 2.27) conditions, *t*(92) = 0.42, *p* = 0.679, *d* = 0.04. To test whether the number of correctly recalled words differed between conditions over time, we conducted a mixed analysis of variance (ANOVA) with recall time (immediate, after 12–24 min, after 7 days) and condition (wakeful resting, d2) as within-subject factors and order of experimental conditions (first wakeful resting-then d2; first d2-then wakeful resting) as a between subjects factor.

**Figure 2:**
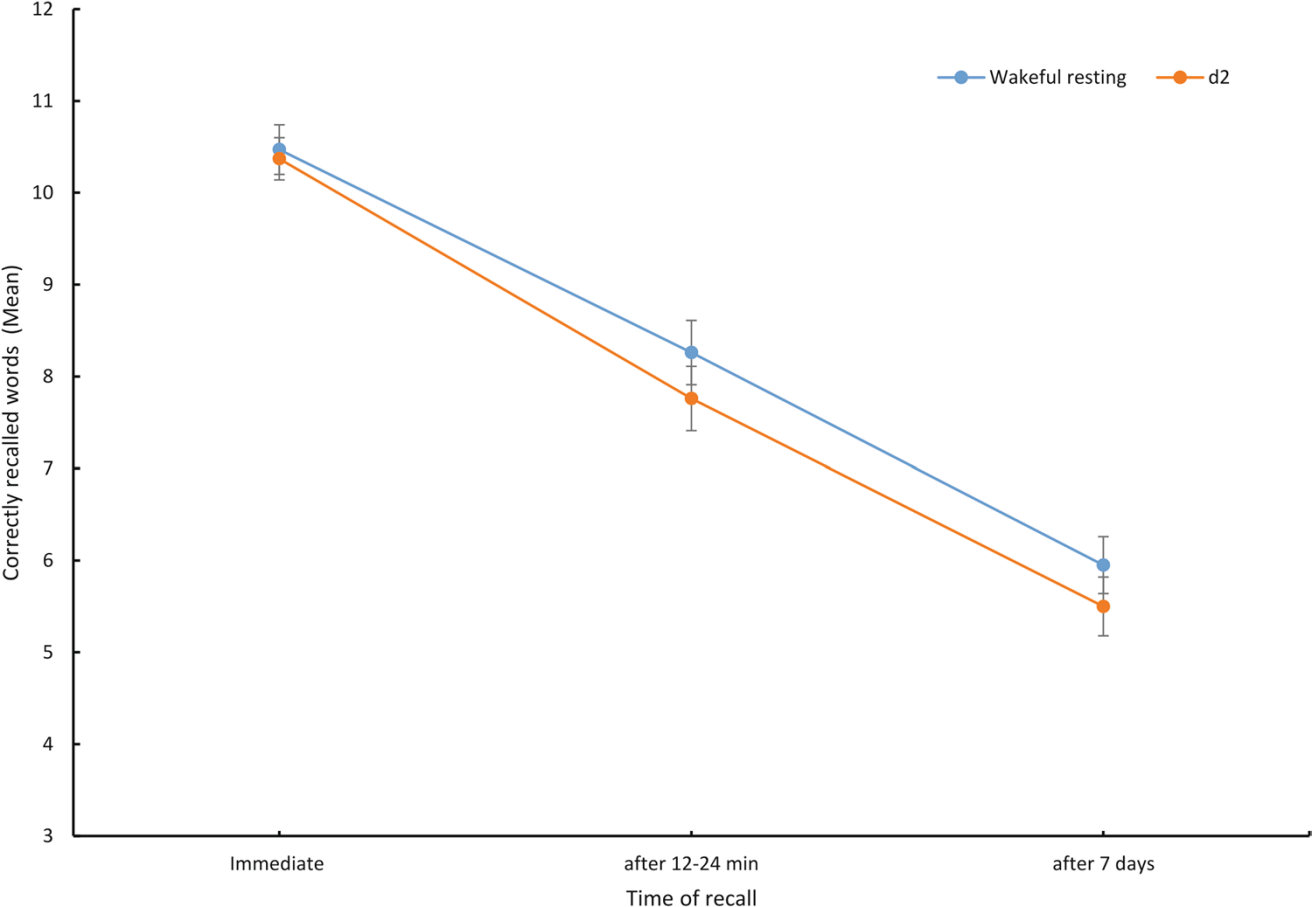
Mean correctly recalled words plotted separately for time of recall (immediate, after 12–24 min, after 7 days) and condition (wakeful resting, d2). Error bars depict standard errors of the mean.

Results revealed a significant main effect of time, *F*(2, 182) = 389.76, *p* < 0.001, *ηp*^*2*^ = 0.81, indicating that memory performance decreased over time (Fig. 2). The main effect of condition was nonsignificant, *F*(1, 91) = 1.06, *p* = 0.306, *ηp*^*2*^ = 0.01, suggesting that memory performance was similar between the wakeful resting and d2 conditions. The time*condition interaction was nonsignificant, *F*(2, 182) = 1.02, *p* = 0.364, *ηp*^*2*^ = 0.01, indicating that memory performance in the wakeful resting condition and the d2 condition decreased similarly over time (Fig. 2). There was no main effect of order (see Supplement 1 online for in-depth analyses regarding order of experimental conditions).

### Inclusion of WMC as covariate

To test whether differences in the number of correctly recalled words between conditions over time were affected by WMC, we conducted a repeated measures analysis of covariance (ANCOVA) with recall time (immediate, after 12–24 min, after 7 days) and condition (wakeful resting, d2) as within-subject factors, order of experimental condition (first wakeful resting-then d2; first d2-then wakeful resting) as between subjects factor and mean centered WMC scores as a covariate. Since alpha error rates for effects not involving the covariate are inflated in repeated measure ANCOVA^58^, effects not involving the covariate should be evaluated based on the ANOVA model reported above. Effects regarding order of delay conditions should therefore be interpreted based on the results presented for the ANOVA model in Supplement 1 online.

**Figure 3:**
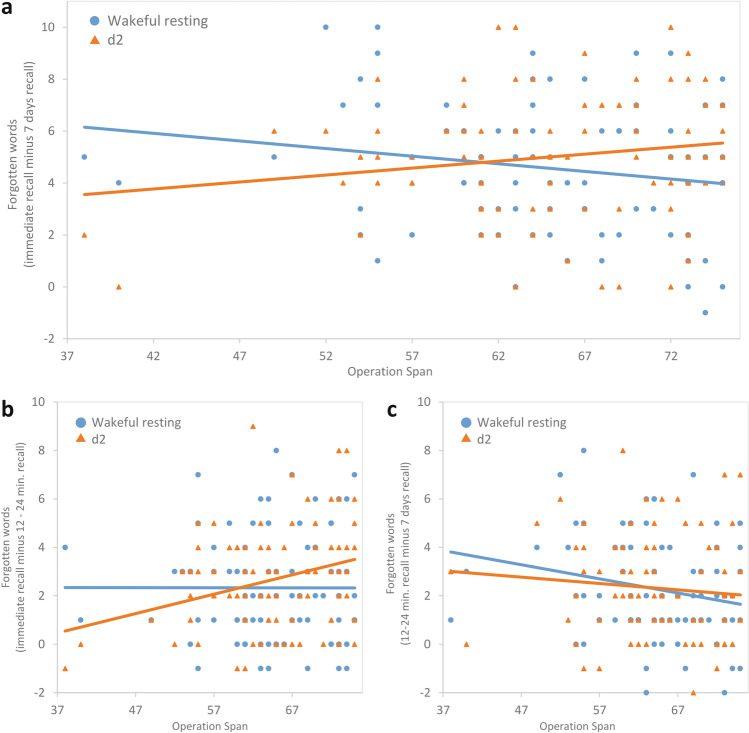
(**a**) Number of forgotten words over the complete retention interval from immediate to second delayed recall after 7 days (y-axis) as a function of WMC (operation span; x-axis) plotted separately for the wakeful resting condition (blue circles) and d2 condition (orange triangles). Panels (**b**) and (**c**) show more detailed partitions of the overall effect (**a**), contrasting the number of forgotten words over retention intervals including the intermediary recall between (**b**) immediate recall and first delayed recall after 12–24 min and (**c**) first delayed recall after 12–24 min and second delayed recall after 7 days.

The time*WMC interaction, *F*(2,180) = 2.53, *p* = 0.083, *ηp*^*2*^ = 0.03 and the delay condition*WMC interaction, *F*(1,90) = 1.52, *p* = 0.221, *ηp*^*2*^ = 0.02, were non-significant. The time*condition*WMC interaction was significant, *F*(2,180) = 4.93, *p* = 0.008, *ηp*^*2*^ = 0.05. These results indicate that participants’ WMC affected differences in memory performance between the wakeful resting condition and d2 condition over retention time (Fig. 3).

To break down these interactions, we performed planned contrasts comparing delayed-recall scores (12–24 min, 7 days) with the immediate recall. The three-way interaction between recall time*condition*WMC was significant for the interval from the immediate to the 12–24 min recall, *F*(1,90) = 5.02, *p* = 0.028, *r* = 0.22 (Fig. 3b) and further increased in strength when looking at the interval between immediate to the 7-day recall, *F*(1,90) = 8.98, *p* = 0.004, *r* = 0.31 (Fig. 3a). These findings suggest that WMC had a moderating effect on the impact of wakeful resting compared to performing the d2 task on the retention of a word list over 7 days.

Exploring the time period between the 12–24 min and 7 day delayed recall tests, we conducted a similar mixed ANCOVA as above, except that for the recall time factor we included only the two delayed recall tests (after 12–24 min, after 7 days). During the time interval between first and second delayed recall the time*WMC interaction was significant, showing that participants with higher WMC retained more words over the 7 day period between delayed recall tests, *F*(1,90) = 6.94, *p* = 0.010, *r* = 0.27. The condition*WMC interaction did not reach significance, *F*(1,90) = 3.29, *p* = 0.073, *r* = 0.19, but inspection of Fig. 3c shows that WMC might have a more positive effect on memory performance in the wakeful resting condition compared to the d2 condition. The time*condition*WMC interaction was not significant over this interval, *F*(1,90) = 0.66, *p* = 0.440, *r* = 0.09.

To examine how many words participants forgot in each condition (wakeful resting, d2) over the three retention intervals shown in Fig. 3a–c, we computed difference scores of the numbers of correctly recalled words for each participant for each retention interval. Exploratory post-hoc Pearson correlations between WMC scores and the number of forgotten words (a) from immediate recall to second delayed recall after 7 days showed that higher WMC individuals forgot less words in the wakeful resting condition (*r* = − 0.22, *p* = 0.031), but not in the d2 condition (*r* = 0.15, *p* = 0.145); (b) from immediate to the first delayed recall after 12–24 min WMC was not related to the number of forgotten words in the wakeful resting condition (*r* = − 0.02, *p* = 0.855), but participants with higher WMC forgot more in the d2 condition (*r* = 0.266, *p* = 0.010); (c) from the first delayed recall after 12–24 min to the second delayed recall after 7 days higher WMC participants forgot less words in the resting condition (*r* = − 0.25, *p* = 0.015), but WMC was not correlated with the number of forgotten words in the d2 condition (*r* = − 0.10, *p* = 0.351).

Twenty-one participants reported that they had expected the surprise free recall test in Session 2. Adding the expectation of the surprise free recall test as a between-subjects factor to the ANCOVA model did not change the results and did not show significant interactions with the previously-described variables (*p* > 0.319). Thus, recall expectancy does not appear to have moderated the sustained memory promotion and its interaction with WMC.

### Mental activity during the delay phase

Rehearsal rates were low and did not differ between the wakeful resting condition (*M* = 1.84, *SD* = 1.31) and d2 condition (*M* = 2.05, *SD* = 2.58), *t*(92) = − 0.75, *p* = 0.457, *d* = 0.10. There was no correlation of active rehearsal and WMC scores, either in the wakeful resting condition, *r* = 0.09, *p* = 0.378, nor in the d2 condition, *r* = − 0.15, *p* = 0.141. Exploratory analysis of the post-condition questionnaire showed that, participants spent more time mind wandering during the wakeful resting condition, while, in the d2 condition, they were more task focused. There were no correlations between WMC and any item measuring mental activity during the wakeful resting condition or the d2 condition (*p* > 0.31). More detailed analyses regarding post learning mental activity and its interaction with memory retention are presented in Supplement 2 online.

## Discussion

In the present study, we investigated whether WMC moderates the effect of a brief period of wakeful resting compared to performing a selective attention task subsequent to encoding a word list. Our results showed that post-encoding manipulations differently affected participants’ memories of a word list, but that this assumption only held true if variance in WMC was accounted for. These results partly support the majority of existing studies showing a beneficial effect of a brief period of wakeful resting immediately after learning compared to task related cognition^6–8,10–12^. Our study extends those findings, showing that interindividual differences in WMC play an important moderating role in the explanation of variation in the effectiveness of wakeful resting compared to task related activity.

Memory consolidation processes following active encoding proceed off-line, during both wake and sleep, without the need for conscious intent, effort or awareness^59^. The resting effect heavily bases on the view that phases in which the brain is not explicitly processing external stimuli, promote memory consolidation processes. Accordingly, any external information that is explicitly processed, in our study the d2 task, should have detrimental effects on memory retention^3,17^. Contrary to many of the findings described above, in six experiments Varma et al.^14^ and in two experiments, Martini et al.^13^ could not find the resting effect—even when several central parameters were manipulated such as distractor task difficulty and content. We initially failed to replicate the memory-supporting effect of the wakeful resting condition compared to a condition of focused attention as well. In the present study, we intended to involve participants in a task that required ongoing focused attention, with few possibilities for task-induced breaks and minimal memory processing demands. We expected to find higher interfering effects of our attention task compared to wakeful resting on memory retention—independent of whether we controlled for WMC or not. Our results revealed that similar amounts of words were retained over shorter (12–24 min) and longer (7 days) retention intervals in both conditions. However, our finding that WMC moderates the wakeful resting effect extends existing assumptions and shows that the effect of wakeful resting in contrast to task related cognition on memory retention might depend on interindividual differences in mental abilities of participants. In our sample, variance between individuals, explained by WMC, is a necessary factor in finding and understanding the influence of post encoding attentional engagement on memory consolidation.

Over a retention interval of 7 days, we found a cross over interaction between the two delay conditions and WMC. The effect of the delay conditions on memory retention is opposite, depending on WMC. An increase in WMC led to an increase in memory retention for the wakeful resting condition along with a decrease in memory retention for the distractor condition. This indicates that high WMC as well as low WMC individuals were more sensitive to the experimental conditions. While high WMC individuals benefitted more from a wakeful resting condition after learning and were more affected by distraction, low WMC individuals showed the opposite effect. Conflicting results described above, that did or did not find a beneficial effect of post encoding wakeful resting, could also be a result of this crossover interaction.

While over the 12–24 min retention interval during Session 1 we only found that WMC was related to lower memory retention in the d2 condition. Over the following 7 day retention interval between Session 1 and 2, we found generally higher memory retention for higher WMC individuals. In line with the findings of Fenn and Hambrick^45,46^, our results support that WMC-related differences during encoding may influence subsequent memory consolidation. While Fenn and Hambrick^45^ were able to show superior memory performance change over a period of sleep compared to daily activity for higher-WMC individuals, our results suggest a similar effect over several minutes of wakeful resting compared to task-focused activity. Resting state connectivity during periods of wakeful resting immediately after learning correlates with improved sleep-dependent memory consolidation^60^. This suggests that memory improvements via reactivation in periods of wakeful resting has the potential to bias or ‘tag’ information for later consolidation processes during sleep^61^.Fenn and Hambrick^45^ speculated that higher WMC individuals might have created stronger associations of learned word pairs during initial acquisition, which might be more likely to be reactivated during offline periods, resulting in greater long-term memory retention. They further hypothesised that higher WMC individuals might have higher baseline spindle activity during sleep and, in turn, derive greater benefit from sleep. Support for this idea was found by an EEG sleep study, showing that participants with higher baseline memory performance (good learners) showed a higher activity and density of sleep spindles indicating a stronger offline reactivation^47^. Furthermore, increased spindle activity during sleep was found in relation with intelligence and general mental ability^62,63^, concepts highly correlated with WMC^64^.

In our study, we did not collect neurophysiological measures. However, based on the neurophysiological findings described above, we speculate that similar processes could lead to the individual differences found in our study. Specifically, we assume that higher WMC individuals more efficiently set up the encoded information during wakeful resting, which in the following nights of sleep led to better memory consolidation in higher-WMC individuals resulting in an increased memory performance over the long term.

In concert with increased memory retention for the wakeful resting condition, we found, that WMC had the opposite effect on memory retention in the d2 condition, especially over the short retention period of 12–24 min. Possibly, because higher WMC individuals might have been more task focused. Several studies showed that higher WMC correlates with controlled attention, in favour of goal-directed information processing^65,66^. According to this “executive attention” view of WM, higher order abilities of higher WMC individuals are caused by lower-order attentional control processes^67^. Post-learning distraction might hinder memory retention in higher WMC individuals more, due to more “efficient” attentional control processes and a stronger focus on the d2 task. Thus, the d2 task might have distracted more resources from memory consolidation in higher WMC individuals, compared to lower WMC individuals, who might allocate less executive resources on the d2 task. This hypothesis is supported by studies showing that higher load, due to performing an additional attention task, only increased proactive interference on memory retention of word lists for high WMC individuals^68^ or findings, that memory retention in lower WMC individuals compared to higher WMC individuals is less affected by a mental context change subsequent to learning^69^.

Our result further suggest, that for lower WMC individuals the resting effect seems to be reversed, in that distraction subsequent to learning is associated with greater memory retention when the participants were performing the d2-task compared to wakefully resting. It is thus possible to speculate that, since lower WMC individuals experience more moment-to-moment fluctuations in arousal than higher WMC individuals^70^, an arousal change induced by the post-learning delay condition led to a reversal of the resting effect in lower WMC individuals. Similar to studies showing that arousal, induced by physical activity^71^ or background white noise^72^, had the potential to enhance memory performance, lower WMC individuals’ memory retention might have benefitted more from a post-learning task related activity than a low arousing resting state.

Higher WMC individuals and lower WMC individuals may apply different memory retention strategies to maintain newly acquired information. Higher WMC individuals might have rehearsed words during the wakeful resting condition more often than lower WMC individuals did, while lower WMC individuals might have rehearsed more during the d2 condition. However, the survey responses suggest that very few participants actively rehearsed the words, that rehearsal did not significantly differ between the wakeful resting and d2 conditions, and that active rehearsal did not correlate with WMC. Furthermore, the expectation of the delayed recall test did not affect retention scores, nor did it show any interactions with WMC. These results indicate that active rehearsal played a minor role for memory retention in this experiment. This is in line with a recent study of Dewar et al.^50^ showing that memory retention of difficult-to-rehearse nonsense words (e.g. phiefnierds) was improved by a brief period of wakeful resting after encoding compared to a spot-the-difference task. However, our rehearsal measure cannot clarify which words, how often and when during the wakeful resting phase the words had been rehearsed. Therefore, our assumptions regarding rehearsal have to be taken cautiously.

Since there was no interaction between WMC and overall differences in word recall, if the factor recall time was not considered, it is safe to assume that the relationship is specific to a change in memory retention due to the experimental manipulation of post-learning activity. These results are in line with those of Fenn and Hambrick^45^, who also found no correlations between WMC and initial recall performance. Even though the three-way interaction between recall time, condition, and WMC showed only small effect sizes over the time course of the first session (12–24 min), over the 7-day retention interval, differences between wakeful resting condition and d2 condition and the moderating influence of WMC on this effect were more pronounced.

In the present study, only a single complex span task was used to measure WMC. Thus, differences in WMC scores might contain variance from both WMC as well as task-specific variance unrelated to this ability, such as speed at solving math problems^56^. Future research should address this issue by replicating our results using a composite or factor score of multiple indicators of WMC.

We cannot rule out that the 12–24 min recall was an additional modulating factor on memory retention. Previous studies have found mixed results on the effects of an intermediary recall on memory retention over the long term. For instance, while Dewar et al.^7^ found no effect of an intermediary recall on memory retention over 7 days, Martini et al.^73^ found an effect. Future studies have to clarify the role of intermediary recalls on the retention of new memories in the context of post-encoding wakeful resting, distraction and WMC.

Finally, an important issue to consider is that, while WMC was a moderating factor for the interaction of post-learning activity and memory consolidation over time, the shape of this interaction might be dependent on different parameters, such as sample characteristics, stimulus material, distractor task or number and time of delayed recall tests. Further studies are required to test the generalisability of our findings under different conditions.

To conclude, our results indicate that WMC is a moderating factor for the resting effect. The impact of a brief period of wakeful resting versus performing a selective attention task subsequent to encoding increased with higher WMC and showed a reversed effect with lower WMC. These results extend existing findings showing that WMC seems not only to modulate sleep-related but also wakeful declarative memory consolidation.

## Supplementary information

Supplementary Information.

## Data Availability

The datasets generated and/or analysed during the current study are available from the corresponding authors on reasonable request.
