# Supplementary material for: Individual differences in working memory capacity moderate effects of post-learning activity on memory consolidation over the long term
Source: Sci Rep. 2020 Oct 21;10:17976. doi: 10.1038/s41598-020-74760-z (PMC7578020; doi:10.1038/s41598-020-74760-z)
Supplement: Supplementary file 1 — Supplementary Information. [file 41598_2020_74760_MOESM1_ESM.docx]

**Individual differences in working memory capacity moderate effects of post-learning activity on memory consolidation over the long term**

Markus Martini^1*†^, Robert Marhenke^1*†^, Caroline Martini^1^, Sonja Rossi^2^, Pierre Sachse^1^

^1^ University of Innsbruck

^2^ Medical University of Innsbruck

† both authors contributed equally

* Corresponding author:

Markus Martini, University of Innsbruck, Innrain 52, 6020 Innsbruck, Austria; [markus.martini@uibk.ac.at](mailto:markus.martini@uibk.ac.at)

Robert Marhenke, University of Innsbruck, Innrain 52, 6020 Innsbruck, Austria; [robert.marhenke@uibk.ac.at](mailto:robert.marhenke@uibk.ac.at)

**Supplementary Material**

**Supplement 1: In-depth analyses regarding order of experimental conditions**

The here described results are supplementary to the results of the ANOVA-analyses of memory performance without WMC as a covariate. In-depth analyses of interactions regarding order of conditions are shown here.

There was no significant main effect of order, *F*(1,91) = .004, *p* = .95, *ηp²* < .01. The time*order interaction was not significant, *F*(2,182) = .54, *p* = .585, *ηp²* < .01. There was a significant interaction of condition*order (Supplementary Figure 1), *F*(1,91) = 22.30, *p* < .001, *ηp²* = .19 as well as a significant interaction of time*condition*order, *F*(2,182) = 9.79, *p* < .001, *ηp²* = .09, indicating that order of the experimental conditions did affect differences in memory retention between experimental conditions. Planned contrasts comparing delayed recall scores with the immediate recall, revealed that over the time period from immediate recall to the delayed recall after 7 days the three-way interaction of time*condition*order was not significant, *F*(1,91) = 1.32, *p* = .253, *r* = .12. Over the 12-24 minutes interval, between immediate and first delayed recall, participants retained less words in whichever condition was performed first compared to the condition performed second (see Supplementary Figure 2), *F*(1,91) = 9.53, *p* = .003, *r* = .31.

These results indicate that there is no overall effect of order of the delay condition on memory retention, but there is a crossover interaction. The effect of order of the delay condition on memory retention is opposite, depending on whichever delay condition was performed first.

**Supplementary Figure 1.** Mean number of words retained as a function of the order of the delay conditions.

**Supplementary Figure 2.** Difference between the number of forgotten words over the retention interval from immediate recall to first delayed recall after 12 – 24 minutes as a function of the order of delay conditions.

**Supplement 2: Mental activity during the delay phase**

See supplementary Table 1 for descriptive statistics of the following analyses. During the wakeful resting condition, participants spent more time mind wandering while, in the d2 condition, they were more task focused. Exploratory post-hoc analysis showed that, within the d2 condition, participants forgot more words over the period from immediate to the first delayed recall when they spent less time thinking about the past, *r* = -.21 *p* = .041, or future, *r* = -.38 *p* < .001 or reported spending less time letting their mind wander, *r* = -.34 *p* = .001. Over the period of 7 days, only ‘thinking about the future’ during the d2 test remained significantly correlated with the number of forgotten words, *r* = -.25, *p* = .015. During wakeful resting, there was no significant correlation between the number of forgotten words over Session 1 and any answers to items assessing mental activity. Over the 7 day retention interval from immediate to the second delayed recall there was a correlation between the number of forgotten words and the amount of time participants reported spending rehearsing the words they learned before, *r* = -.22, *p* = .037.

| Supplementary Table 1 | | | | | | | | | | |
| --- | --- | --- | --- | --- | --- | --- | --- | --- | --- | --- |
| *Means, standard deviation and t-test statistics of questionnaire responses regarding mental activity during either the wakeful resting or d2 condition.* | | | | | | | | | | |
|  | Wakeful resting | | | | d2 | | Difference between delay conditions | | | |
| Post-condition questionnaire | | *M* | *SD* | *M* | | *SD* | | *t*(92) | *p* | *d* |
| Thinking about the past | | 3.08 | 1.16 | 2.03 | | 1.32 | | 7.22 | <.001 | .85 |
| Thinking about the future | | 3.61 | 1.19 | 2.07 | | 1.18 | | 10.49 | <.001 | 1.35 |
| I let my mind wander | | 4.40 | 1.28 | 2.14 | | 1.34 | | 12.50 | <.001 | 1.73 |
| My mind was blank | | 1.76 | 1.19 | 2.80 | | 1.83 | | -4,87 | <.001 | -.67 |
| I've been thinking about what I'm doing right now | | 2.53 | 1.45 | 3.63 | | 1.79 | | -5.20 | <.001 | -.67 |
| I tried to rehearse the words I had learned before | | 1.84 | 1.31 | 2.05 | | 2.59 | | -.75 | .457 | -.10 |

*Note. M* = Mean; *SD* = standard deviation.
